# The variant c.274A>G (p.Asn92Asp) in *KRT17* in a patient with pachyonychia congenita and a novel clinical feature of acne inversa

**DOI:** 10.3389/fgene.2024.1365581

**Published:** 2024-11-13

**Authors:** Huaiyu Wang, Changhua Zhu, Linxin Dong, Baofeng Wu, Jingjing Liu, Lihang Lin, Daoyao Lin, Xiangqi Chen, Xuemin Xiao

**Affiliations:** ^1^ Department of Dermatology, The Union Hospital, Fujian Medical University, Fuzhou, China; ^2^ MyGenostics Inc., Beijing, China; ^3^ Department of Dermatology, 900Th Hospital of Joint Logistics Support Force, Fuzhou, China

**Keywords:** pachyonychia congenita, acne inversa, KRT17 gene, mutation, whole-exome sequencing, Notch, PI3K, AKT

## Abstract

**Introduction:**

The occurrence of pachyonychia congenita (PC) and acne inversa (AI) may be related to gene mutations. The aim of this study is to identify the genetic cause in a patient with PC and AI, and to explore the possible molecular mechanism of their co-occurrence.

**Methods:**

The clinical data of the proband were collected, and the genomic DNA of the proband and unaffected parents were extracted. The variant sites of the proband were identified by whole-exome sequencing, and then the variant sites of the proband and his parents were verified by Sanger sequencing.

**Results:**

A heterozygous variant in *KRT17* gene was found in the patient, resulting in a missense amino acid variant (p.N92D). The variant was not found in his parents or 100 unrelated healthy controls. In addition, this variant was not found in the gnomad v4 database. The three-dimensional structure analysis of the protein suggested that the polarity of amino acids changed after the variant. After lentiviral plasmid transfection into HaCaT cells, the expression level of NOTCH signaling decreased in the constructed c.274A>G (p.Asn92Asp) of *KRT-17* mutant cells compared to that in the wild-type. Subsequent verification confirmed that differences in the expression levels of p-PI3K, AKT and p-AKT between the groups were not statistically significant.

**Discussion:**

Although this variant has been reported previously, our findings could expand the spectrum of co-occurrence of PC and AI with *KRT17* gene variants, and elucidated the possible pathogenesis at the protein level, thereby laying a foundation for the genetic diagnosis and genetic counseling provided to individuals with PC.

## 1 Introduction

Pachyonychia congenita (PC) is an autosomal dominant hereditary disease with rich clinical phenotypic heterogeneity. The exact prevalence of PC is approximately 0.9 cases per million people (Pinna et al., 2019). The phenotypic spectrum includes painful palmoplantar keratoderma (PKK), nail/toenail hypertrophy, neonatal or prenatal teeth, multiple lipoblastomas, oral mucosal leukoplakia, and follicular hyperkeratosis (Pavlovsky et al., 2021). Hypertrophic nail dystrophy, characterized by thickened and discolored fingernails and toenails, is a common clinical feature of PC. PC is caused by a mutation in five keratin genes (*KRT6A, KRT6B, KRT6C, KRT16,* and *KRT17*) (Samuelov et al., 2020). Genetic sequencing has allowed for insights into the pathophysiology of the disease and variations depending on the subtype and nature of mutations. PC-*K17* and PC-*K6A* cause an earlier onset of nail disease than the other subtypes, with the majority of patients having symptoms within the first year of life, whereas subtypes PC-*K16* and PC-*K6B* cause the largest range of nail symptoms (Wu and Lipner, 2021).

Acne inversa (AI), also known as the follicular occlusion triad, includes hidradenitis suppurativa, acne conglobata, and perifolliculitis capitis abscedens et suffodiens. AI has a multifactorial pathogenesis, such as genetic susceptibility, hormonal dysregulation, microbiome dysregulation, and immune dysregulation (Jenkins et al., 2023). In addition to sporadic forms, familial forms have been reported in approximately 40% of patients (Goldburg et al., 2020). AI is a chronic inflammatory disease of the skin that causes painful subcutaneous nodules, abscesses, sinus, and scar formation. Inflammation in patients with AI is not restricted to the skin but is systemic, affecting several other organs (Sabat et al., 2020). The most important genes involved in the pathogenesis of AI are those encoding the γ-secretase subunits (1p21.1–1q25.3) and the gene encoding *PSTPIP1* (proline threonine-phosphatase interacting protein 1) (15q24.3) (Gao et al., 2006). To date, 78 γ-secretase gene mutations have been reported in acne inversion patients and their families, of which 52 were in *NCSTN*, 22 in *PSENEN*, 3 in *PSEN1*, and only 1 likely pathogenic variant in *APH1B* (Ratnamala et al., 2023). A growing body of evidence suggests that the initial events in AI with γ-secretase gene mutations are hyperkeratosis of terminal hair follicle infundibular keratinocytes and resulting follicular occlusion, leading to rupture of the hair follicle, with subsequent inflammatory responses and possible secondary infection (Zhou et al., 2021). *PSTPIP1* mutations have been detected in autoinflammatory syndromes associated with HS, namely, PASH (pyoderma gangrenosum, acne, and hidradenitis suppurativa) syndrome and PAPASH (pyogenic arthritis, pyoderma gangrenosum, acne, and Hidradenitis suppurativa) syndrome (Nikolakis et al., 2021).

Although it has been reported that these two diseases are concurrent, the pathogenesis is still unclear. We reported a case of PC accompanied with AI and detected its pathogenic gene to explore its pathogenesis in molecular genetics.

## 2 Materials and methods

### 2.1 Patient data collection

This study collected the clinical data of a proband with PC and AI and his seven unaffected family members. At the same time, laboratory tests such as blood routine, urine routine, C-reactive protein, liver function, renal function, blood lipid, onychomycosis culture, dermoscopy, and skin biopsy were performed on the proband. This study was approved by the Ethics Committee of the Union Hospital of Fujian Medical University. All participants provided written informed consent.

### 2.2 Whole-exome sequencing

Approximately 2 mL of peripheral blood (EDTA anticoagulant) was collected from the patient and the patient’s parents. A blood genomic DNA extraction kit (Kangwei Century, China) was used to extract genomic DNA from the sample, and the NanoDrop (2000) instrument (Thermo Fisher, United States) was used to analyze the extracted nucleic acids. Genomic libraries were prepared using a standard library construction kit (self-developed by Mackinac), and the libraries were analyzed by NanoDrop (2000) and agarose gel electrophoresis. Using the GenCap liquid phase capture target gene technology (Maginot, China), 723 genes related to skin diseases were captured. The HiSeq 4000 (Illumina) sequencing platform was used for double-ended sequencing. The original sequencing data were removed from contamination and coupling sequences, and the filtered sequences were aligned to the human genome reference sequence (HG19) in the NCBI database using the BWA software (http://bio-bwa.sourceforge.net/). All SNPs (single-nucleotide polymorphisms) and INDELs (insertions and deletions) were analyzed and annotated using the GATK and ANNOVAR software. Missense variants were predicted for pathogenicity and conservation using PolyPhen-2, GERP+, SIFT, and MutationTaster software, and cleaved site changes were analyzed for pathogenicity using Spidex software.

### 2.3 Sanger sequencing

The candidate variant sites obtained by analysis and screening were verified by Sanger sequencing, and the genotype of the parents was determined. The sequencing primers were forward primers: 5′-CTG​CCA​GCT​CAA​TTG​CC-3’ and reverse primers: 5 ‘- TAT​AAG​GAG​AGC​GGC​GGA​AC-3.’ Amplification was performed via polymerase chain reaction (PCR) using the following conditions: pre-denaturation at 98°C for 2 min, denaturation at 98°C for 10 s, annealing at 55°C–65°C for 30 s, and extension at 72°C for 10 s for 35 cycles. After 35 cycles, extension at 72°C for 1 min was performed. PCR products were examined via electrophoresis on a 2% agarose gel. The PCR-amplified target fragments were recovered, purified using a PCR purification kit (QIAGEN, Germany), and sequenced using an ABI 3730XL automated sequencer (Applied Biosystems, United States). The sequencing results were compared with those of the *KRT17* (NM_000422.3) gene sequence (genome version GRCh37/hg19), and Mutation Surveyor software was used for analysis. If there were any abnormalities, reverse sequencing was performed.

### 2.4 Protein conservation and 3D structure predictions

KRT17 protein sequences were obtained from the National Center of Biotechnology Information (NCBI) (https://www.ncbi.nlm.nih.gov/). The three-dimensional structure of *KRT17* was obtained from the AlphaFold Protein Structure Database (https://alphafold.ebi.ac.uk/). We predicted the structure of the mutant using I-TASSER (https://zhanglab.ccmb.med.umich.edu/I-TASSER/), and PyMOL was used to analyze and visualize the model by referring to Version 7.6.

### 2.5 Construction and identification of KRT17 wild-type and c.274A>G recombinant lentiviral vectors

**FIGURE 1:**
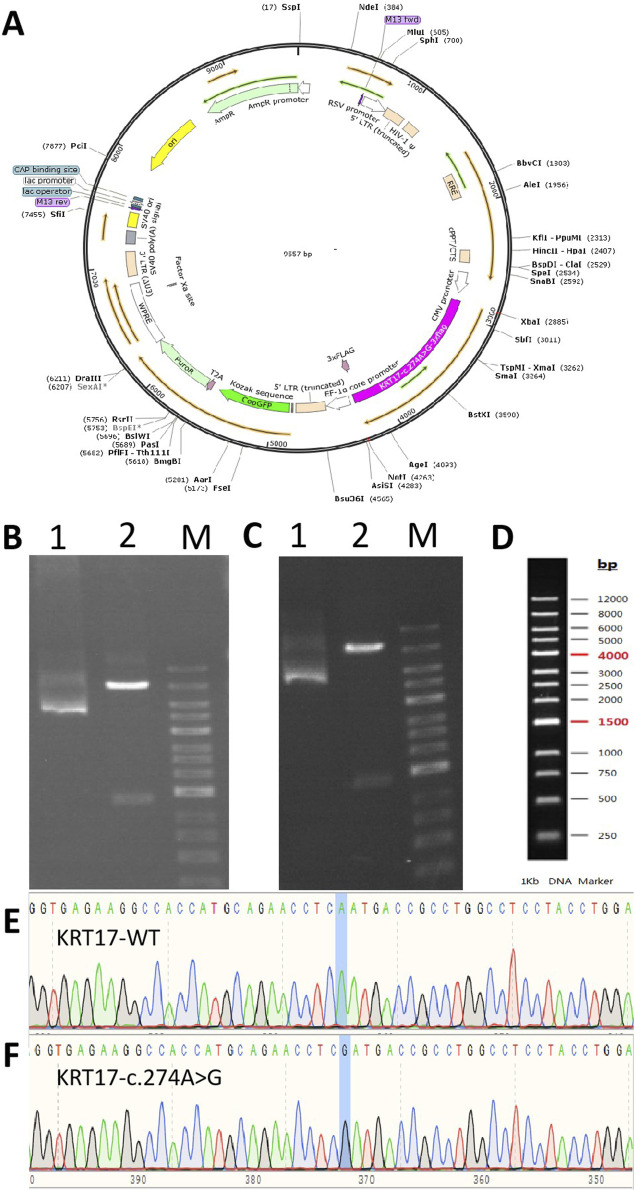
Gene cloning and plasmid vector construction. **(A)** Schematic diagram of the mutant c.274A>G plasmid vector: pCDH-CMV-KRT17 (c.274A > G)-EF1-copGFP-T2A-Puro. **(B)** Electropherogram of the wild-type plasmid digested using NotI/XhoI. **(C)** Electropherogram of the mutant c.274A>G plasmid digested using NotI/XhoI. Lane 1: undigested electropherogram, lane 2: electropherogram after double digestion, and lane 3: DNA marker. **(D)** DNA marker of **(B, C)**. **(E, F)** Sequencing of the clone plasmid of the *KRT17* gene.

The plasmid synthesis scheme is shown in Figure 1. The expression vector used was pLVZG-CMV-MCS-EF1-copGFP-T2A-Puro to synthesize the *KRT17* gene with double restriction sites: XbaI/NotI (Figures 1B–D). Next, various mutant plasmids were constructed as follows: wild-type plasmid 1 and pLVZG-CMV-KRT17 (WT)-3xflag-EF1-copGFP-T2A-Puro with double restriction sites XbaI/NotI and mutant plasmid 2 and pLVZG-CMV-KRT17 (c.274A>G)-3xflag-EF1-copGFP-T2A-Puro with double restriction sites XbaI/NotI, encompassing the 274A>G variant site of *KRT17*. Amplification, validation, and sequencing of the target genes were performed. Human embryonic kidney 293 cell line 293FT cells were used to package plasmids into *Lentivirus*, the cell culture supernatant produced within 72 h was collected using a sterile filter, and then the virus concentrated and purified. Recombinant lentiviral vectors KRT17 (c.274A>G) and its negative control KRT17 (WT) were constructed by the third-generation lentiviral packaging system (Zolgene Biotechnology, China).

### 2.6 Lentivirus transfection and generation of stable cells

Human immortalized keratinocyte line HaCaT cells (CTCC, United States) were cultured in Dulbecco’s modified Eagle’s medium with high glucose containing 10% fetal bovine serum (Thermo Fisher Scientific, United States). Well-grown HaCaT cells were seeded into six-well plates at 1 × 10^5^ per well and cultivated overnight to reach approximately 50% confluency. Cells were then cultured in a serum-free medium containing 5 μg/mL polybrene (Zolgene, China) at 37°C and 5% CO2 and infected with recombinant lentiviral vectors at a multiplicity of infection (MOI) of 40. After 4–6 h, the medium was replaced with the fresh serum-containing medium. After 72 h of post-infection, cells were cultivated in the medium containing 0.5 μg/mL puromycin (Sigma) for 2 weeks to screen and obtain stable cell lines, which were then cultured in bulk for subsequent assays.

### 2.7 Quantitative real-time PCR

Total RNA was isolated from HaCaT cells using the TRNzol Universal REAGENT (TIANGEN, China). Reverse transcription and qRT-PCR were used to detect differences in the transcript levels of KRT17. cDNA was synthesized from the total RNA using FastKing gDNA Dispelling RT SuperMix (TIANGEN, China). qRT-PCR reactions were performed using BlasTaq™ 2X qPCR MasterMix (ABM, Canada) using cDNA as a template in a reaction system (20 μL): 10 μL BlasTaq™ 2X qPCR MasterMix, 0.5 μL forward primer (10 μmol/L), 0.5 μL reverse primer (10 μmol/L), 2 μL cDNA, and 7 μL RNase-Free ddH2O for 40 cycles. qRT-PCR was performed using the ABI 7500 Real-Time PCR system. The PCR primers were synthesized by Fuzhou Shangya Biotechnology Co., Ltd. The specific sequences were as follows: KRT17, forward 5-GGT​GGG​TGG​TGA​GAT​CAA​TGT-3′ and reverse 5′-CGC​GGT​TCA​GTT​CCT​CTG​TC-3′ and β-actin, forward 5-TCA​CCC​ACA​CTG​TGC​CCA​TCT​ACG​A-3′ and reverse 5'-CAG​CGG​AAC​CGC​TCA​TTG​CCA​ATG​G-3 '. The relative expression levels of KRT17 were calculated by the 2^−ΔΔCT^ method and normalized to β-actin.

### 2.8 Western blotting assay

Total protein from HaCaT cells was cultured, lysed (RIPA lysis buffer), and extracted, and the protein expression was detected. Protein samples and marker were loaded into electrophoresis gel wells, wet-transferred to polyvinylidene fluoride (PVDF) membranes after electrophoresis, and soaked in 5% albumin from bovine serum (BSA) at room temperature for 2 h. The membranes were washed once, incubated using primary antibody solutions (KRT17 [ab109725, Abcam, United Kingdom], 1:1,000; Notch1 [ab52627, Abcam, United Kingdom], 1:1,000; Notch2 [D76A6, CST, United States], 1:1,000; Notch3 [D11B8, CST, United States]; AKT [51077-1-AP, Proteintech, United States]; Phospho-AKT [Ser473] [28731-1-AP, Proteintech, United States], 1:1,000; PI3K p85 alpha [AF6241, Affinity, United States], 1:1,000; Phospho-PI3K [AF3242, Affinity, United States],1:1,000; and β-actin [ab8226, Abcam, United Kingdom], 1:1,000), and diluted in 5% bovine serum albumin (BSA) overnight at 4°C. After washing the membranes three times, 5% BSA was added to dilute the horseradish peroxidase-labeled secondary antibody, and the samples were incubated in a shaker at room temperature for 2 h. The sections were visualized after three washes with TBST. The target bands were finally detected using a Thermo ECL Kit (34080, Thermo Fisher). Tanon-5200Multi (Tanon) was used for exposure imaging. Protein expression was analyzed using ImageQuant (LAS-4000, Fujifilm) software. Bands were quantified using ImageJ software.

## 3 Results

### 3.1 Family members

An 18-year-old male presented with multiple evident cysts of approximately 0.3–0.7 cm diameter over his face, neck, and scrotum, but without ulceration, bleeding, or other symptoms. The onset of skin lesions began after birth. When he was 4 months old, the fingernails and toenails thickened and gradually deformed, showing an opaque yellowish brown color on the deck. Five years ago, he suffered from repeated episodes of papules, nodules, pustules, and cysts on the back. After that, the skin lesions worsened with the development of red papules, nodules, and abscesses on the head and face, as well as bilaterally in the armpits, groin, and buttocks. The most severe involvement was in the axilla with pain, and the Hurley classification was II. His parents did not share a consanguineous relationship, and there were no other individuals in the family with similar symptoms.

**FIGURE 2:**
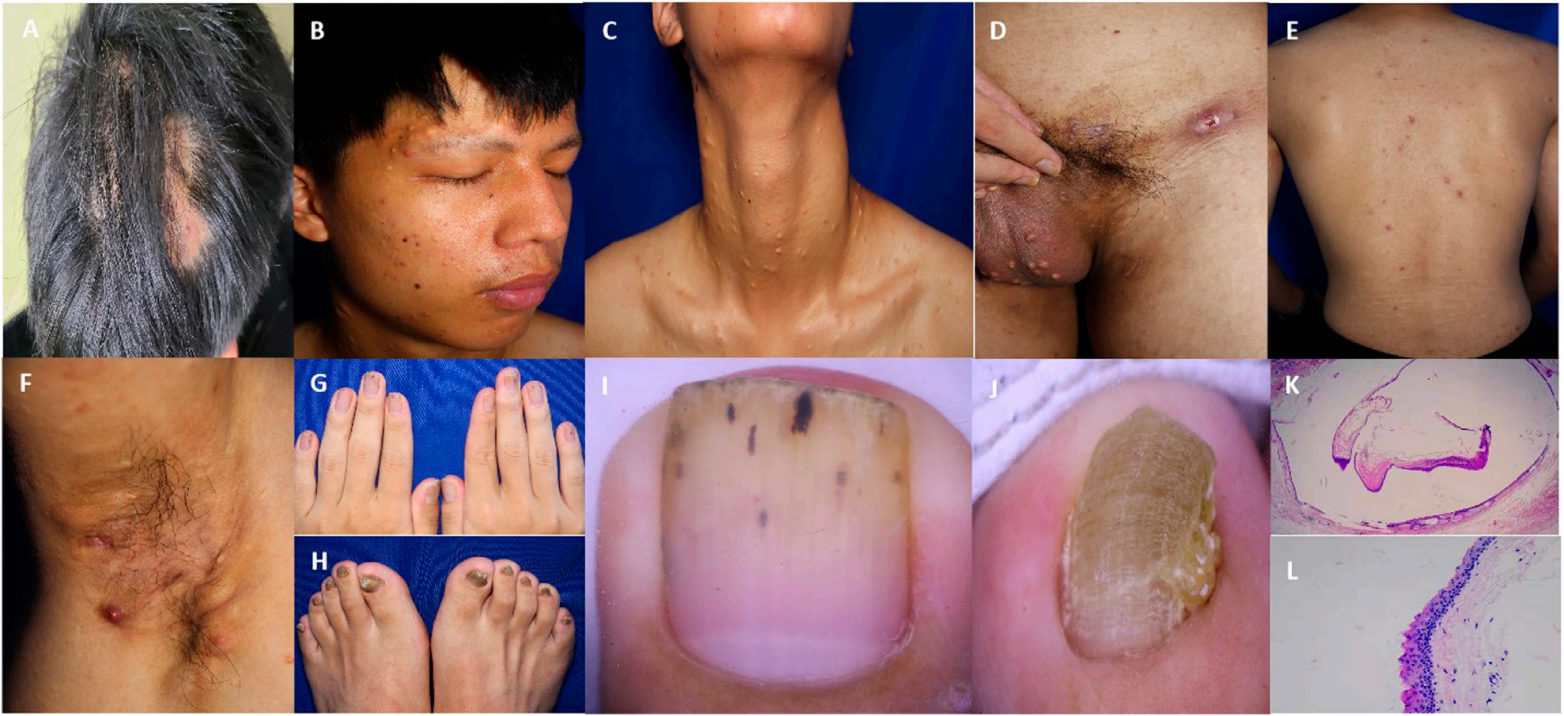
Clinical and pathological features of the patient. **(A)** Multiple papules and nodules on the scalp with oval alopecia spots around. **(B–D)** Steatocystoma multiplex lesions over the face, neck, and scrotum. **(E)** Papules and pigmentation on the back. **(F)** Inflammatory papules, nodules, pustules, and sinuses in the axilla. **(G, H)** Thickening and deformation of the fingernail and toenail. **(I)** Nail dermoscopy showed the thickening of the nail plate with black punctate and linear structures. **(J)** Nail dermoscopy showed that the toenail plate was significantly thickened, with obvious transverse grooves and longitudinal ridges. **(K)** The cyst wall contained a cavity formed by a thin layer of the epithelium, with sebaceous material in the cavity. (25x HE) **(L)** An eosinophilic wavy epidermis was seen on the cyst wall, with a serrated arrangement of endothelial cells (200x HE).

He had a body mass index of 20.2. Skin examination revealed papules, pustules, and flake alopecia on the head (Figure 2A). Numerous cysts were present over his face (Figure 2B), neck (Figure 2C), and scrotum (Figure 2D). Multiple black comedones, inflammatory papules, and hyperpigmentation were seen on his back (Figure 2E). Nodules and cysts with discharged pus and scars were present over the axilla (Figure 2F) and inguinal folds (Figure 2D). The patient’s toenails and fingernails were markedly thickened. The toenails were severely affected, and the bunions of both feet were claw-like (Figures 2G, H).

Laboratory examinations showed that the white blood cell count was 11.60 × 10^9^/L (reference value 4.00 × 10^9^–10.00 × 10^9^/L), the percentage of neutrophils was 83.20% (reference value 50.00%–70.00%), and C-reactive protein was 10.43 mg/L (reference value 0–8.00 mg/L). No obvious abnormality was found in other examinations. The microscopic examination and culture of onychomycetes were negative. Nail dermoscopy showed thickened nails, transverse grooves and longitudinal ridges of toenails, and black punctate and linear structures of fingernails (Figures 2I, J). The skin tissue pathology revealed that the intradermal cyst structure is seen, the eosinophilic wavy epidermis can be seen on the cyst wall, and the endothelial cells are arranged in a serrated shape, indicating steatocystoma multiplex (SM) (Figures 2K, L).

### 3.2 *KRT17* gene variants and Sanger sequencing validation

**FIGURE 3:**
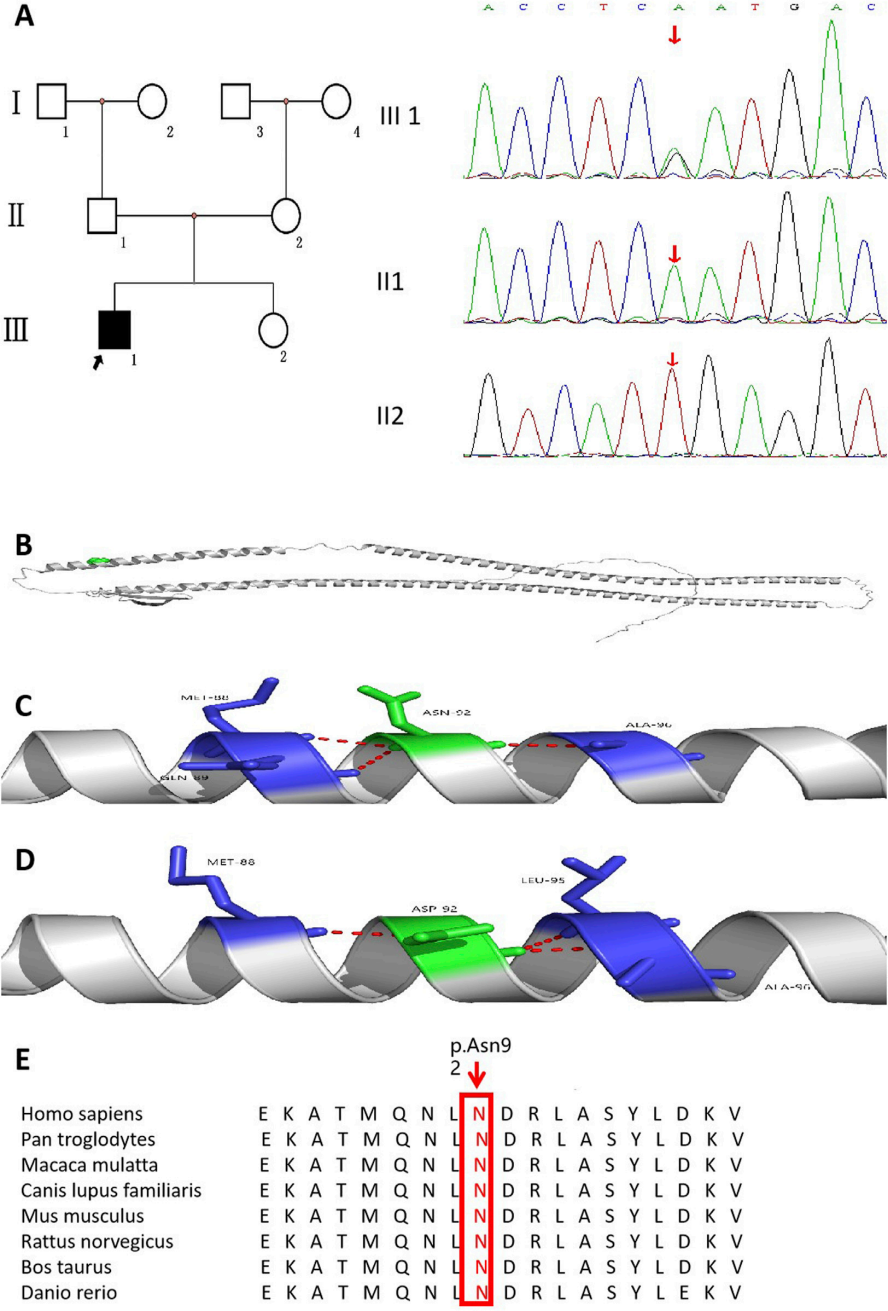
Pedigree of the family affected by PC accompanied with AI. **(A)** Family pedigree of the proband. **(B)** KRT17 protein structure (cartoon model, gray) and the distribution of variant site 92 on the protein structure (green sphere, located in the α-helix region). **(C)** is the 3D partial enlarged view of the WT KRT17 protein. The Asn92, respectively, forms a hydrogen bond with the Met88, Gln89, and Ala96. **(D)** is the 3D partial enlarged view of the mutant KRT17 protein. When Asn is replaced by Asp, the hydrogen bond interaction with Gln89 disappears, and a new hydrogen bond interaction is formed with Leu95. **(E)** Alignment of KRT17 protein sequences from different species.

The results of whole-exome sequencing indicated that the proband had a heterozygous missense variant in the *KRT17* gene, which was a change of nucleotide 274 from adenine A to guanine G (c.274A>G), resulting in the change in the 92nd amino acid from asparagine to aspartic acid (p.N92D). The variant was not found in his parents or 100 unrelated healthy controls (Figure 3A). It was also not found in the gnomAD v4 database, the NHLBI Exome Sequencing Project, or the Exome Aggregation Consortium browser. This supports the idea that this is a *de novo* causative variant rather than a polymorphism. The results of short tandem repeat (STR) analysis indicated that 18 of the 24 STR loci in the proband were heterozygous, and two loci at the same locus were from his father and mother, supporting the biological relatedness of the proband to his parents.

### 3.3 Bioinformatics analyses of the identified variant

This variant has been reported in ClinVar. According to the ACMG guidelines, this variant was preliminarily determined to be pathogenic (PS2 + PS4 + PM1 + PM2_Supporting + PM5 + PP3_Strong): 1) PS2: this *de novo* (both maternity and paternity confirmed) variant has been reported in a patient with the disease and no family history. 2) PS4: this variant has been reported in a large population where it was shown to co-segregate with pachyonychia congenita in multiple affected individuals (McLean et al., 1995). 3) PM1: the variant was located in the mutation hotspot region (helix initiation motif). 4) PM2_Supporting: this variant was absent from controls in Exome Sequencing Project, 1,000 Genomes Project, gnomAD database, or Exome Aggregation Consortium. 5) PM5: many other pathogenic variants in patients with pachyonychia congenita have been reported at the same residues (c.275A>C,p.Asn92Thr; c.275A>G,p.Asn92Ser; and c.274A>C,p.Asn92His) according to the Human Gene Mutation Database (Ofaiche et al., 2014; Zhang et al., 2020; Smith et al., 1997). 6) PP3_Strong: the prediction results of bioinformatics protein function comprehensive prediction software REVEL, SIFT, PolyPhen_2, MutationTaster, and GERP+ indicated harm. We searched the relative protein position of the patient’s variant in the gene bank and found that Asn92 is a conserved amino acid residue in *Homo sapiens*, *Pan troglodytes*, *Macaca mulatta*, *Canis lupus* familiaris, *Mus musculus*, *Rattus norvegicus*, *Bos taurus*, and *Danio rerio* (Figure 3E). The 3D structure analysis of KRT17 showed that in the wild-type, Asn92 formed hydrogen bond interaction with amino acids Met88, Gln89, and Ala96. When Asn was replaced by Asp, the amino acid at position 92 changed from neutral to negative, and the hydrogen bond with Gln89 disappeared, forming a new hydrogen bond interaction with Leu95 (Figures 3B–D). This may have affected the structure and function of the protein.

### 3.4 Expression of *KRT17*-WT and mutant in HaCaT cells

**FIGURE 4:**
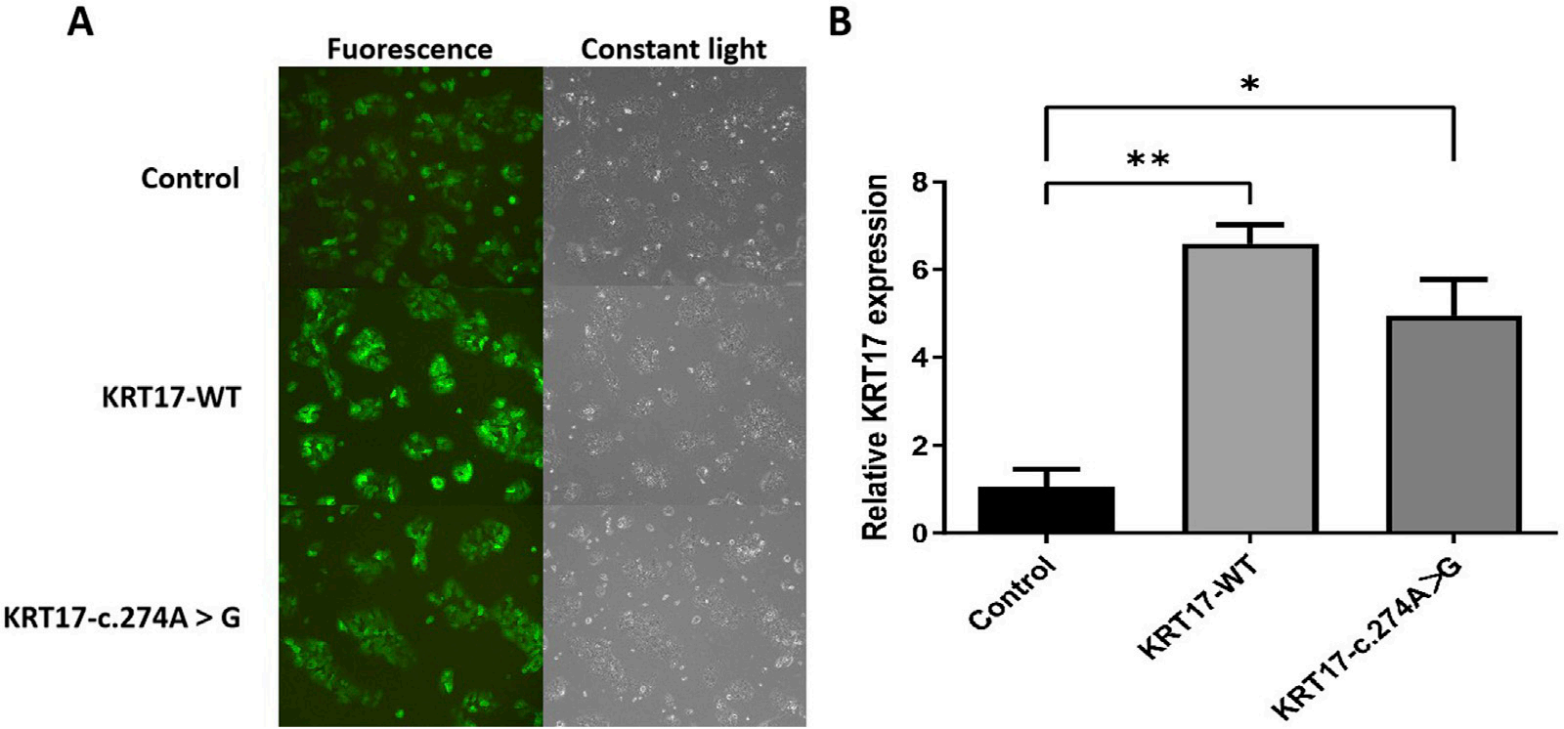
Gene cloning and plasmid vector construction. **(A)** Green fluorescent protein expression after the transfection of cells carrying unloaded (control), wild-type (*KRT17*-WT), and c.274A>G mutants, suggesting that there was no significant difference in transfection efficiency among the groups. **(B)** Relative *KRT17* mRNA expression levels (*, *P* < 0.05 and **, *P* < 0.01) in HaCaT cells by control, *KRT17*-WT, and c.274A>G mutant.

After 72 h of infection with ZVE1003 cells grown in the logarithmic phase with pCDH lentiviral empty load, pLVZG-CMV-*KRT17* (WT)-3xflag-EF1-copGFP-T2A-Puro, and pLVZG-CMV-*KRT17* (c.274A>G)-3xflag-EF1-copGFP-T2A-Puro lentiviral expression vectors, the cells highly expressed green fluorescent protein (Figure 4A), indicating good growth status. Relative *KRT17* mRNA expression levels of the *KRT17*-WT and c.274A>G mutant of the *KRT17* gene in HaCaT cells were examined via qRT-PCR. Green fluorescent protein was used as an external reference to compare the relative differences in the *KRT17* mRNA expression group, and the results indicated that *KRT17* mRNA expression was downregulated in c.274A>G mutants cells compared to that in control group (P < 0.05) (Figure 4B).

### 3.5 Western blotting to verify protein expression

**FIGURE 5:**
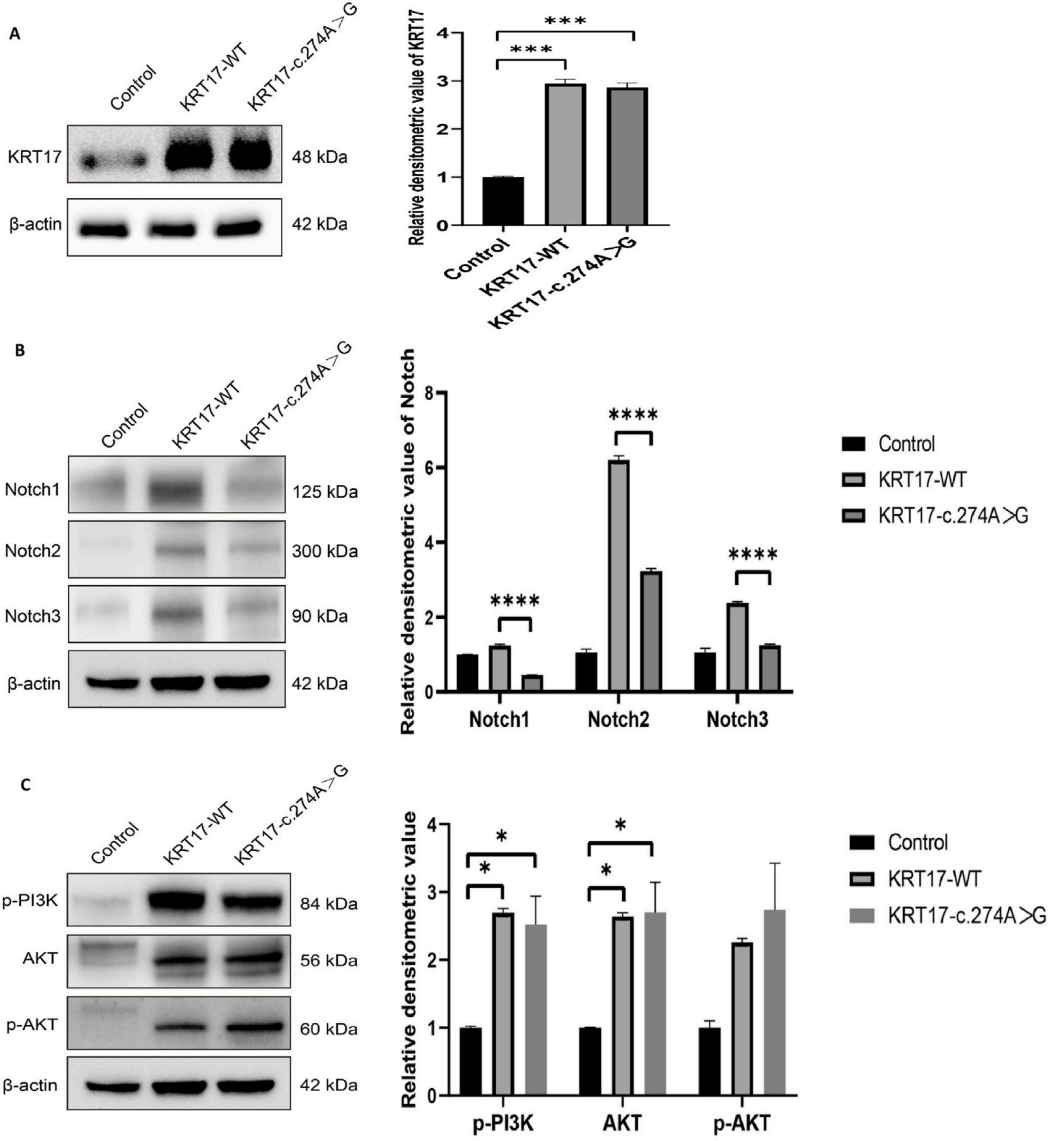
Results of protein expression: **(A)** Protein expression levels of wild-type KRT17 expression vector and mutant KRT17 expression vector containing the c.274A>G variant in HaCaT cells; β-actin is used as a loading control. Western blot showing increased protein levels of KRT17-WT and KRT17-c.374A>G. **(B)** Western blot showing reduced protein levels of NOTCH. **(C)** p-PI3K, AKT, and p-AKT protein expressions were unchanged after silencing KRT for 72 h in HaCaT cells (*, *P* < 0.05, ***, *P* < 0.01, and ****, *P* < 0.0001).

Compared with the negative control group, the protein levels of KRT17-WT and KRT17-c.274A>G transfected groups increased, which was statistically significant (*P* < 0.01), and there was no difference between the two transfected groups (Figure 5A). The transfection of the KRT17 variant-encoding vector resulted in a significant reduction in NOTCH activity compared with the wild-type vector (*P* < 0.0001) (Figure 5B), indicating that the KRT17-induced variant resulted in a reduction in NOTCH activity. p-PI3K and p-AKT are the phosphorylation products of PI3K and AKT, respectively. In HaCaT cells transfected with plasmids, the protein levels of AKT were not significantly different from those of the wild-type group, and the protein levels of p-PI3K and p-AKT were not significantly different from those of the wild-type group (*P* > 0.05) (Figure 5C), indicating that the PI3K–AKT signaling pathway was not activated.

## 4 Discussion

PC is a rare autosomal dominant genetic skin disorder that is divided into two subtypes: PC-1 (Jadassohn–Lewandowski type) and PC-2 (Jackson–Lawler type), in which PC-1 is caused by mutations in *KRT6A* or *KRT16* and is characterized by oral leukoplakia. In contrast, PC-2 is caused by *KRT6B* or *KRT17* mutations and is characterized by cysts and viviparous teeth (Feinstein et al., 1998; Paller et al., 1991; Stieglitz and Centerwall, 1983; Su WP et al., 1990). The *KRT16* p.L132P mutation was significantly associated with younger age of onset, the presence of palmar keratoderma oral leukokeratosis, and a higher number of involved nails (Samuelov et al., 2021). The *KRT17* p.L99P mutation resulted in an increased number of involved fingernails and patients demonstrating 20-nail dystrophy (Samuelov et al., 2021). It was previously suggested that the p.K354N mutation in *KRT16* and the p.N109D mutation in *KRT17* are associated with delayed presentation of clinical manifestations in PC (Connors et al., 2001; Smith, 2004; Xiao et al., 2004).

AI is a chronic inflammatory disease, which mainly affects the skin folds in the axilla, groin, buttocks, and perianal region. The inflammation usually starts around the hair follicles, gradually evolves into painful nodules and abscesses, and subsequently may result in sinus development and scarring (Wolk et al., 2020). Predisposing factors include genetic factors, obesity, and smoking. More than one-third of the patients have a family history. Recently, mutations in *KRT17* have been reported in PC and HS as well as PC with follicular occlusion tetrad (Pavlovsky et al., 2022; Musumeci et al., 2019). Even with those results, the *KRT17* role in PC accompanied with AI remains debated for the lack of sufficient evidence.

Here, we found a reported disease-causing variant in the KRT17 gene c.274A>G (p.Asn92Asp) of this patient, which further supports the diagnosis of PC. We also identified a phenotype for which this variant has not been reported. The patient presented with thickened and deformed nails and steatocystoma multiplex. Nail dermoscopy showed thickening of nails and toenails. It is consistent with the diagnosis of PC Ⅱ. At the same time, he had several inflammatory papules, nodules, and pustules on the head, axilla, back, groin, and buttocks, some of which were broken with pus and formed sinus tracts, which was consistent with the diagnosis of AI. The patient did not have a mutation in γ-secretase on genetic sequencing, and he was of normal figure and had no smoking or family history. It has been found that Asn92 in KRT17 is completely conserved in intermediate filaments, and mutations in its rod domain interfere with the early or higher-order stages of fiber assembly, which can lead to fiber abnormalities (IFs) (McLean et al., 1995). Previous studies have found that the *KRT17* mutation is a genetic marker for PC II and familial SM (Ghosh et al., 2017; Wang J et al., 2018). KRT17 regulates a myriad of biological processes, including cell proliferation and growth, skin inflammation, and hair follicle cycle (Yang et al., 2019). It has been suggested that KRT17, which could shuttle in and out of the nucleus due to the presence of nuclear localization signals and nuclear export signals, plays a key role in promoting the cell-autonomous expression of genes encoding inflammatory and immune cytokine effectors (Hobbs et al., 2016). It has been speculated that the distribution of KRT17 in pilosebaceous glands and glandular structures may be responsible for cyst formation in PC-2 (Oh et al., 2008). A study has suggested that AI is related to the activation of skin keratinocytes (KCs), leading to the regulation of keratin deposition and the stimulation of autologous inflammatory diseases (Giamarellos-Bourboulis et al., 2022). In our study, a heterozygous mutation of the *KRT17* gene was found by gene sequencing, resulting in the change in amino acid 92 from asparagine to aspartic acid (p.N92D). The expression of *KRT17* increased significantly after *KRT17* mutation. At the same time, the 3D structure analysis showed that the *KRT17* protein was neutral in the wild type. The side chain of the patient changed after Asn > Asp at position 92, resulting in the change in the amino acid from neutral to negative charge and from polar neutral to acidic amino acid. Therefore, this variant will change the local interaction network of the protein, which may lead to the dysfunction of the protein and the occurrence of diseases. Basic biochemical foundations such as hydrophobic contacts, electrostatic interactions, hydrogen bonding, steric hindrance, and physicochemical amino acid properties are important for correct dimer, tetramer, and higher-order oligomer assembly of IFs (Wu TT et al., 2021). We hypothesized that the *KRT17* gene mutation may lead to the change in amino acid properties, affect the correct assembly of IFs, and thus affect the function of keratin, leading to the fragile, hyperproliferative, and undifferentiated state of keratinized epithelium, resulting in corresponding skin changes. This demonstrates that *KRT17* should be considered a gene associated with PC comorbid with AI.

Regarding the pathogenic mechanisms, the protein keratin is encoded by the *KRT*, which is the main structural protein that forms the IF cytoskeletal network in epithelial cells. Keratin is divided into type I and type II and plays an important role in maintaining the integrity of the epidermis in addition to regulating cell growth and migration (Wu TT et al., 2021). Keratin 17 belongs to the intermediate family of type I and is mainly present in epithelial appendages, such as hair follicles, sebaceous glands, and other glands (Tong and Coulombe, 2004). The EGFR/ERBB subfamily and phosphoinositide 3-kinase (PI3K)/AKT signaling pathway play essential roles in maintaining the homeostasis of keratinocytes and epidermis (Zhou et al., 2021). The PI3K/AKT signaling pathway is related to cell proliferation and differentiation, and the activation of the PI3K/AKT pathway in mouse skin induced the proliferation of progenitor cells in interfollicular epidermis and hair follicles, leading to follicular and epidermal hyperplasia (Hessam et al., 2021). The NOTCH signaling pathway exerts a crucial role in regulating and maintaining skin homeostasis, orchestrating keratinocyte differentiation at the level of interfollicular epidermis and hair follicles, and finally contributing to epithelial barrier formation (Condorelli et al., 2021). The previous study has shown that the coexistence of PC and AI is related to the NOTCH signaling pathway, but this has not been validated at the protein level (Pavlovsky et al., 2022). Therefore, for the first time, we verified NOTCH and PI3K/AKT signaling pathways at the protein level.

Our study showed that NOTCH expression was significantly reduced after transfection of the *KRT17* variant coding vector, suggesting that *KRT17* mutation may affect NOTCH signaling. NOTCH signaling is a highly conserved, ubiquitous, cell–cell communication pathway involved in cell fate, proliferation, and tissue homeostasis both in embryonic development and adult life (Siebel and Lendahl, 2017). Notch proteins comprise a family of four type 1 transmembrane receptors, including the four genes Notch1, Notch2, Notch3, and Notch4. Notch signaling functions as a molecular switch that controls the transition of cells between skin layers during the epidermal differentiation process (Osamura et al., 2005). It was speculated that the decreased expression of NOTCH molecules induced the dysfunction of NOTCH signaling and played an important role in the coexistence of two diseases. The *KRT17* variant may inhibit NOTCH signaling, leading to hair follicle cycle arrest, hair follicle conversion into a sac, and inhibition of sebaceous gland differentiation, making the patient present with both AI and PC (Watt et al., 2008). The *KRT17* gene may regulate the common pathogenic pathway between PC Ⅱ and AI.

## 5 Concluding remarks

In summary, the pathogenesis of PC II with AI is unknown, and the type and severity of the lesion that occurs in a patient depend on the specific keratin gene mutation, in which tissues the abnormal keratin is expressed and the local biochemical changes caused by the mutation. In addition, the phenotypes of the *KRT17* gene mutations are very diverse, including nail, skin, and oral mucosa manifestations. Therefore, the missense variant c.274A>G (p.Asn92Asp) in the *KRT17* gene identified in this study may reflect the different expression profiles caused by a common genetic change. This may be related to the downregulation of the NOTCH pathway caused by the *KRT17* mutation. This study enriches the spectrum of the coexistence of PC and AI. Importantly, we validated the relevant pathways at the protein level. The mechanism underlying the co-occurrence of PC and AI is not fully understood; further molecular biological research of the *KRT17* mutation and the introduction of *in vivo* models with genetic deficiency are warranted.

## Data Availability

The original contributions presented in the study are publicly available. This data can be found here: https://ngdc.cncb.ac.cn/gsa-human/, HRA009091.

## References

[B1] Condorelli A. G. El Hachem M. Zambruno G. Nystrom A. Candi E. Castiglia D. (2021). Notch-ing up knowledge on molecular mechanisms of skin fibrosis: focus on the multifaceted Notch signalling pathway. J. Biomed. Sci. 28(1):36. 10.1186/s12929-021-00732-8 33966637 PMC8106838

[B2] Connors J. B. Rahil A. K. Smith F. J. McLean W. H. Milstone L. M. (2001). Delayed-onset pachyonychia congenita associated with a novel mutation in the central 2B domain of keratin 16. Br. J. Dermatol. 144 (5), 1058–1062. 10.1046/j.1365-2133.2001.04199.x 11359398

[B3] Feinstein A. Friedman J. Schewach-Millet M. (1998). Pachyonychia congenita. J. Am. Acad. Dermatol 19 (4), 705–711. 10.1016/s0190-9622(88)70226-1 3053803

[B4] Gao M. Wang P. G. Cui Y. Yang S. Zhang Y. H. Lin D. (2006). Inversa acne (hidradenitis suppurativa): a case report and identification of the locus at chromosome 1p21.1-1q25.3. J. Invest. Dermatol. 126 (6), 1302–1306. 10.1038/sj.jid.5700272 16543891

[B5] Ghosh R. Chatterjee K. Barua J. K. Roy A. (2017). Cutaneous cysts with nail dystrophy in a young female: a classical association. Indian J. Dermatol 62 (6), 661–664. 10.4103/ijd.IJD_473_16 29263544 PMC5724318

[B6] Giamarellos-Bourboulis E. J. (2022). Modulation of keratin deposition and pathogenesis of hidradenitis suppurativa: evidence coming from pachyonychia congenita. Br. J. Dermatol. 187 (5), e170–e171. Epub 2022 Aug 22. 10.1111/bjd.21807 35996837

[B7] Goldburg S. R. Strober B. E. Payette M. J. (2020). Hidradenitis suppurativa: epidemiology, clinical presentation, and pathogenesis. J. Am. Acad. Dermatol. 82 (5), 1045–1058. Epub 2019 Oct 9. 10.1016/j.jaad.2019.08.090 31604104

[B8] Hessam S. Gambichler T. Skrygan M. Scholl L. Sand M. Meyer T. (2021). Increased expression profile of NCSTN, Notch and PI3K/AKT3 in hidradenitis suppurativa. J. Eur. Acad. Dermatol Venereol. 35 (1), 203–210. Epub 2020 Nov 5. 10.1111/jdv.16962 32978818

[B9] Hobbs R. P. Jacob J. T. Coulombe P. A. (2016). Keratins are going nuclear. Dev. Cell. 38(3):227–233. 10.1016/j.devcel.2016.07.022 27505414 PMC5511689

[B10] Jenkins T. Isaac J. Edwards A. Okoye G. A. (2023). Hidradenitis suppurativa. Dermatol Clin. 41 (3), 471–479. Epub 2023 Apr 15. PMID: 37236715. 10.1016/j.det.2023.02.001 37236715

[B11] McLean W. H. Rugg E. L. Lunny D. P. Morley S. M. Lane E. B. Swensson O. (1995). Keratin 16 and keratin 17 mutations cause pachyonychia congenita. Nat. Genet. 9 (3), 273–278. 10.1038/ng0395-273 7539673

[B12] Musumeci M. L. Fiorentini F. Bianchi L. Cascella R. Giardina E. Caputo V. (2019). Follicular occlusion tetrad in a male patient with pachyonychia congenita: clinical and genetic analysis. J. Eur. Acad. Dermatol Venereol. 33 (Suppl. 6), 36–39. 10.1111/jdv.15851 31535756

[B41] NanoDrop (2000). Instrument. United States: Thermo Fisher.

[B13] Nikolakis G. Kaleta K. P. Vaiopoulos A. G. Wolter K. Baroud S. Wojas-Pelc A. (2021). Phenotypes and pathophysiology of syndromic hidradenitis suppurativa: different faces of the same disease? A systematic review. Dermatology. 237 (5), 673–697. Epub 2020 Sep 17. 10.1159/000509873 32942279

[B14] Ofaiche J. Duchatelet S. Fraitag S. Nassif A. Nougué J. Hovnanian A. (2014). Familial pachyonychia congenita with steatocystoma multiplex and multiple abscesses of the scalp due to the p.Asn92Ser mutation in keratin 17. Br. J. Dermatol. 171 (6), 1565–1567. Epub 2014 Nov 2. 10.1111/bjd.13123 24842198

[B15] Oh A. C. Jones B. Liao H. Smith F. J. Solomon R. Egan C. A. (2008). Recurrent mutation in keratin 17 in a large family with pachyonychia congenita type 2. Arch. Dermatol Res. 300 (5), 211–214. Epub 2008 Mar 18. 10.1007/s00403-008-0840-7 18347808

[B16] Osamura R. Takekoshi S. Kajiwara H. Minematsu T. (2005). Histochemical technologies for genomics and proteomics: laser capture microdissection (LCM) and tissue microarray (TMA). Acta histochem. Cytochem. 38, 185–188. 10.1267/ahc.38.185

[B17] Paller A. S. Moore J. A. Scher R. (1991). Pachyonychia congenita tarda. A late-onset form of pachyonychia congenita. Arch. Dermatol May 127 (5), 701–703. 1827243

[B18] Pavlovsky M. Peled A. Samuelov L. Malki L. Malovitski K. Assaf S. (2021). Molecular epidemiology of pachyonychia congenita in the Israeli population. Clin. Exp. Dermatol. 46 (4), 663–668. Epub 2020 Dec 20. 10.1111/ced.14509 33190296

[B19] Pavlovsky M. Peled A. Sarig O. Astman N. Malki L. Meijers O. (2022). Coexistence of pachyonychia congenita and hidradenitis suppurativa: more than a coincidence. Br. J. Dermatol. 187 (3), 392–400. Epub 2022 Jun 17. 10.1111/bjd.21674 35606927 PMC9796395

[B20] Pinna R. Cocco F. Campus G. Conti G. Milia E. Sardella A. (2019). Genetic and developmental disorders of the oral mucosa: epidemiology; molecular mechanisms; diagnostic criteria; management. Periodontol. 80 (1), 12–27. 10.1111/prd.12261 31090139

[B21] Ratnamala U. Jain N. K. Jhala D. D. Prasad P. V. S. Saiyed N. Nair S. (2023). An updated mutation spectrum of the γ-secretase complex: novel NCSTN gene mutation in an Indian family with hidradenitis suppurativa and acne conglobata. Indian J. Dermatol 68 (2), 141–147. 10.4103/ijd.ijd_995_21 37275792 PMC10238988

[B22] Sabat R. Jemec G. B. E. Matusiak Ł. Kimball A. B. Prens E. Wolk K. (2020). Hidradenitis suppurativa. Nat. Rev. Dis. Prim. 6(1):18. 10.1038/s41572-020-0149-1 32165620

[B23] Samuelov L. Sarig O. Adir N. Pavlovsky M. Smith F. J. Schwartz J. (2021). Identification of clinically useful predictive genetic variants in pachyonychia congenita. Clin. Exp. Dermatol. 46 (5), 867–873. Epub 2021 Mar 17. 10.1111/ced.14569 33486795

[B24] Samuelov L. Smith F. J. D. Hansen C. D. Sprecher E. (2020). Revisiting pachyonychia congenita: a case-cohort study of 815 patients. Br. J. Dermatol 182 (3), 738–746. Epub 2020 Jan 14. 10.1111/bjd.18794 31823354

[B25] Siebel C. Lendahl U. (2017). Notch signaling in development, tissue homeostasis, and disease. Physiol. Rev. 97(4):1235–1294. 10.1152/physrev.00005.2017 28794168

[B26] Smith F. J. (2004). Nail that mutation-keratin 17 defect in late-onset pachyonychia. J. Invest. Dermatol. 122 (4), x–xi. 10.1111/j.0022-202X.2004.22437.x 15102104

[B27] Smith F. J. Corden L. D. Rugg E. L. Ratnavel R. Leigh I. M. Moss C. (1997). Missense mutations in keratin 17 cause either pachyonychia congenita type 2 or a phenotype resembling steatocystoma multiplex. J. Invest. Dermatol. 108 (2), 220–223. 10.1111/1523-1747.ep12335315 9008238

[B29] Stieglitz J. B. Centerwall W. R. (1983). Pachyonychia Congenita (Jadassohn-Lewandowsky syndrome): a seventeen-member, four-generation pedigree with unusual respiratory and dental involvement. Am. J. Med. Genet. 14 (1), 21–28. 10.1002/ajmg.1320140105 6829608

[B30] Su W. P. Chun S. I. Hammond D. E. Gordon H. (1990). Pachyonychia congenita: a clinical study of 12 cases and review of the literature. Pediatr. Dermatol Mar. 7 (1), 33–38. 10.1111/j.1525-1470.1990.tb01070.x 2140447

[B31] Tong X. Coulombe P. A. (2004). A novel mouse type I intermediate filament gene, keratin 17n (K17n), exhibits preferred expression in nail tissue. J. Invest. Dermatol 122 (4), 965–970. 10.1111/j.0022-202X.2004.22422.x 15102087

[B32] Wang J. Li J. Li X. Lei D. Xiao W. Li Z. (2018). A recurrent mutation in the KRT17 gene responsible for severe steatocystoma multiplex in a large Chinese family. Clin. Exp. Dermatol 43 (2), 205–208. Epub 2017 Dec 8. 10.1111/ced.13311 29218738

[B33] Watt F. M. Estrach S. Ambler C. A. (2008). Epidermal Notch signalling: differentiation, cancer and adhesion. Curr. Opin. Cell. Biol. 20 (2), 171–179. Epub 2008 Mar 14. 10.1016/j.ceb.2008.01.010 18342499 PMC2324124

[B34] Wolk K. Join-Lambert O. Sabat R. (2020). Aetiology and pathogenesis of hidradenitis suppurativa. Br. J. Dermatol 183 (6), 999–1010. Epub 2020 Oct 13. 10.1111/bjd.19556 33048349

[B35] Wu A. G. Lipner S. R. (2021). Distinctions in the management, patient impact, and clinical profiles of pachyonychia congenita subtypes. Skin. Appendage Disord. 7 (3), 194–202. 10.1159/000513340 34055907 PMC8138248

[B36] Wu T. T. Eldirany S. A. Bunick C. G. Teng J. M. C. (2021). Genotype‒structurotype‒phenotype correlations in patients with pachyonychia congenita. J. Invest. Dermatol 141 (12), 2876–2884.e4. Epub 2021 Jun 8. 10.1016/j.jid.2021.03.035 34116063 PMC8922998

[B37] Xiao S. X. Feng Y. G. Ren X. R. Tan S. S. Li L. Wang J. M. (2004). A novel mutation in the second half of the keratin 17 1A domain in a large pedigree with delayed-onset pachyonychia congenita type 2. J. Invest. Dermatol. 122 (4), 892–895. 10.1111/j.0022-202X.2004.22408.x 15102078

[B38] Yang L. Zhang S. Wang G. (2019). Keratin 17 in disease pathogenesis: from cancer to dermatoses. J. Pathol. 247 (2), 158–165. Epub 2018 Dec 7. 10.1002/path.5178 30306595

[B39] Zhang B. Sun L. Fu X. Yu G. Liu H. Zhang F. (2020). Mutation analysis of the KRT17 gene in steatocystoma multiplex and a brief literature review. Clin. Exp. Dermatol 45 (1), 132–134. Epub 2019 Sep 17. 10.1111/ced.14030 31237972

[B40] Zhou P. Liu J. Xu T. Guo Y. Han Y. He Y. (2021). Mutations in γ-secretase subunit-encoding PSENEN gene alone may not be sufficient for the development of acne inversa. J. Dermatol Sci. 103 (2), 73–81. Epub 2021 Jun 18. 10.1016/j.jdermsci.2021.06.007 34330582

